# Understanding the Effects of Interfacial Lithium Ion Concentration on Lithium Metal Anode

**DOI:** 10.1002/advs.202104145

**Published:** 2021-12-22

**Authors:** Jimin Park, Son Ha, Jae Young Jung, Jae‐Hwan Hyun, Seung‐Ho Yu, Hyung‐Kyu Lim, Nam Dong Kim, Young Soo Yun

**Affiliations:** ^1^ KU‐KIST Graduate School of Converging Science and Technology Korea University 145, Anam‐ro, Seongbuk‐gu Seoul 02841 South Korea; ^2^ Functional Composites Materials Research Center Korea Institute of Science and Technology (KIST) 92, Chudong‐ro Wanju‐gun Jeollabuk‐do 55324 Republic of Korea; ^3^ Department of Chemical and Biological Engineering Korea University 145, Anam‐ro, Seongbuk‐gu Seoul 02841 South Korea; ^4^ Division of Chemical Engineering and Bioengineering Kangwon National University Chuncheon Gangwon‐do 24341 South Korea; ^5^ Department of Integrative Energy Engineering Korea University 145, Anam‐ro, Seongbuk‐gu Seoul 02841 South Korea

**Keywords:** lithiophilic electrodes, lithium metal anodes, nitrogen‐doped carbon, pseudocapacitive electrodes, solid–electrolyte–interface layer

## Abstract

Despite the development of multidimensional state‐of‐the‐art electrode materials for constructing better lithium metal anodes (LMAs), the key factors influencing the electrochemical performance of LMAs are still poorly understood. Herein, it is demonstrated that the local lithium ion concentration at the interface between the electrode and electrolyte exerts significant influence on the electrochemical performance of LMAs. The local ion concentration is multiplied by introducing pseudocapacitive nanocarbons (PNCs) containing numerous heteroatoms, because PNCs can store large numbers of lithium ions in a pseudocapacitive manner, and promote the formation of an electrochemical double layer. The high interfacial lithium ion concentration induces the formation of lithium‐rich inorganic solid–electrolyte–interface layers with high ionic conductivities, and facilitates sustainable and stable supplies of lithium ion charge carriers on the overall active surfaces of the PNCs. Accordingly, the PNC‐induced LMA exhibits high Coulombic efficiencies, high rate capabilities, and stable cycling performance.

## Graphical abstract

The local lithium ion concentration at the interface between the electrode and electrolyte is a key factor in determining the electrochemical performance of lithium metal anodes. A high interfacial lithium ion concentration induces the formation of lithium‐rich inorganic solid–electrolyte–interface layers with high ionic conductivities, and facilitates sustainable and stable supplies of lithium ion charge carriers.

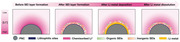

## 1 Introduction

Rapidly advancing modern society is moving toward a hyper‐connected community concatenated with artificial intelligence and data centers through the Internet of Things.^[^ ^1^, ^2^ ^]^ Accordingly, state‐of‐the‐art electronic machineries that consume much more energy and power are becoming pervasive for supporting the advancing society.^[^ ^3^ ^]^ Lithium ion batteries (LIBs) are considered the most promising power source for emerging electronics because of their electrochemical performance, as LIBs afford high round‐trip efficiency, cycling reversibility, and high energy densities (≈250 Wh kg^−1^), and have seen commercial success in the markets of several mobile electronic devices.^[^ ^4^, ^5^ ^]^ However, the theoretical energy densities of conventional LIBs based on intercalation chemistry are now running up against technical limitations. In addition, the sluggish kinetics of lithium ion diffusion in the graphite anode restrict the power capabilities of LIBs.^[^ ^6^, ^7^ ^]^ Hence, new chemistry is needed to overcome the technical barriers hindering the accomplishment of higher energy and power densities.

Recent revisiting studies on lithium metal anodes (LMAs) confirmed that significantly enhanced energy and power densities can be achieved by replacing graphite anodes with LMAs because of the higher theoretical capacity (≈3860 mA h g^−1^) and lower redox potential (−3.04 V vs SHE) of LMAs.^[^ ^8^, ^9^, ^10^ ^]^ A key issue in realizing a feasible LMA is suppressing the uncontrolled growth of metal with a high aspect ratio, called lithium dendrite.^[^ ^8^, ^9^, ^10^, ^11^, ^12^ ^]^ The dendrite grows into a 3D porous structure with a high specific surface area, where a large amount of solid–electrolyte–interface (SEI) layer is formed, accompanied by electrolyte decomposition, throughout the overall active surface area.^[^ ^13^, ^14^ ^]^ Furthermore, the SEI layers are prone to collapse and stack on the electrode surface when the deposited metal is extracted.^[^ ^15^, ^16^ ^]^ Accordingly, large consumption of electrolyte solution and formation of much thicker SEI layers are unavoidable during continuous cycling, which reduces the lithium ion concentration at the interface between the electrode and electrolyte.^[^ ^17^ ^]^ The lack of electrolyte solution, particularly on the local electrode surface, can cause a severe increase in the concentration polarization, accelerating the diffusion‐controlled growth of lithium metal, which induces dendrite formation.^[^ ^18^, ^19^ ^]^ The fatal problem caused by lithium dendrites is manifested as low coulombic efficiency (CE) and poor cycling performance.^[^ ^13^, ^18^, ^19^ ^]^ In addition, sudden cell death can occur due to short‐circuiting induced by dendrite formation, which is responsible for cell explosion.^[^ ^9^, ^11^, ^19^ ^]^ Therefore, enormous research efforts have focused on mitigating the formation of dendritic metal in LMAs.

A noticeable improvement in the electrochemical performance can be achieved by designing (1) 3D‐structured lithiophilic electrode materials (3D‐LEMs) and (2) better SEI layers, having high chemical/mechanical stabilities and high ionic conductivities.^[^ ^20^, ^21^, ^22^, ^23^, ^24^, ^25^, ^26^, ^27^, ^28^, ^29^, ^30^, ^31^, ^32^ ^]^ The high active surface areas and catalytic nucleation sites in 3D‐LEMs can simultaneously cause extensive lithium metal growth on the electrode surfaces, which can remarkably reduce the effective current density, leading to kinetic‐controlled lithium metal deposition. The explosive growth of lithium dendrites in a specific region can be mitigated by inducing lithium ion deconcentration.^[^ ^20^, ^21^, ^22^, ^23^, ^24^, ^25^, ^26^ ^]^ Several studies have also revealed that lithium‐rich inorganic SEI (L–I–SEI) layers such as Li_2_O, Li_2_CO_3_, and LiF can induce uniform lithium ion transport in the interfacial area. The stable L–I–SEI layer can mitigate continuous electrolyte decomposition, even under harsh conditions, with infinite volume expansion, leading to homogeneous lithium metal deposition/dissolution cycles.^[^ ^26^, ^27^, ^28^, ^29^, ^30^, ^31^, ^32^ ^]^ Accordingly, the effects of various electrolyte additives have been actively studied in attempts to form better SEI layers with large numbers of inorganic components, where positive effects have been demonstrated, particularly on the surface of 3D‐LEMs.^[^ ^26^ ^]^ Hence, previous reports suggest that high‐performance LMAs can be realized by a synergistic combination of 3D‐LEMs and L–I–SEI layers. However, despite their effectiveness, fundamental understanding of the key factors determining the high electrochemical performance of LMAs is still elusive. This hinders intensive research toward the unidirectional aim to achieve technological advances, thereby retarding the development of feasible lithium metal batteries.

In this study, we demonstrate that the lithium ion concentration at the interface between the electrode and electrolyte has a major impact on the electrochemical performance of LMAs. For this purpose, three different types of electrode materials (bare Cu foil, carbon black, and pseudocapacitive nanocarbon (PNC)) are applied as LMA materials. Carbon‐based electrode materials with high surface areas can store lithium ions on the surface, where the nitrogen and oxygen heteroatom‐rich PNCs show higher lithium ion chemisorption capacity. The lithium‐covered surface is prone to forming lithium‐rich inorganic compounds upon electrolyte decomposition, leading to L–I–SEI layers with a significantly low film resistance. In addition, electrochemical double layers (EDLs), that is, lithium‐rich ionic layers, are formed around the high‐surface‐area PNC electrode, leading to higher coulombic efficiency (CE), higher rate capabilities, and cycling stability through a sustainable and stable supply of lithium ion charge carriers.

## 2 Results and Discussion

**Figure 1:**
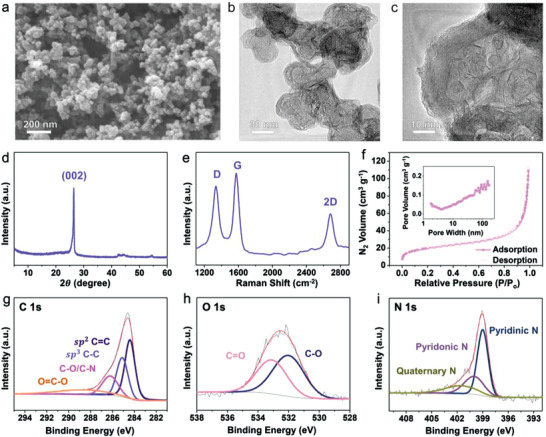
Material properties of PNCs. a) FE‐SEM and b,c) FE‐TEM images at different magnifications. d) XRD pattern, e) Raman spectra, f) nitrogen adsorption and desorption isotherms (inset shows pore size distribution), XPS g) C 1s, h) O 1s, and i) N 1s profiles.

The PNCs comprised spherical nanoparticles with a diameter of ≈30 nm. The particles were randomly aggregated to form a 3D nanoporous structure, as shown in the field‐emission scanning electron microscope (FE‐SEM) and field‐emission transmission electron microscopy (FE‐TEM) images (**Figure** **1**a,b). High‐resolution FE‐TEM images revealed that the PNCs are composed of ordered graphitic nanowalls with large curvatures (Figure 1c; Figure S1, Supporting Information). The sharp peak centered at ≈26.5° in the X‐ray diffraction (XRD) pattern, corresponding to the graphite (002) plane, supports the ordered carbon microstructure of the PNCs (Figure 1d). By applying the Scherrer equation, the thickness (*L*_c_) of the crystalline graphitic domains was calculated as ≈25.3 nm. However, the lateral domain size (*L*_a_) obtained from the characteristic *D* and *G* bands of the Raman spectrum was ≈4.5 nm, which is much smaller than the *L*_c_ value (Figure 1e). This could be due to the intrinsic morphological characteristics with large curvatures and the presence of numerous heteroatom dopants in the polyhexagonal carbon plane. The intrinsic/extrinsic defects are active hosts to store lithium ions by pseudocapacitive manners, wherein the smaller *L*_a_ value is inevitable for the pseudocapacitive electrode materials.^[^ ^33^, ^34^ ^]^ In the strong reduction/oxidation cycles with lithium metal under organic electrolyte, defective carbon surfaces with the smaller *L*c can form thermodynamically more stable byproducts such as CO, CO_2_, and Li_2_CO_3_. In addition, a heteroatom dangling bond linked on active carbon surfaces can deform the planar sp2 carbon ring into sp^3^‐structured stereoscopic structures, which can deteriorate the chemical stability of the carbon building blocks. Thus, the defective carbon structure is prone to be degraded in the repetitive lithium metal deposition–dissolution cycles, leading to insufficient cycling performances. The demerit of no well‐ordered carbon materials in electrochemical systems was confirmed in the previously reported literatures.^[^ ^35^, ^36^, ^37^, ^38^, ^39^ ^]^ However, despite the defective graphenic carbon structure, the thick graphitic stacking with large *L*_c_ value can mitigate the degradation of the carbon structures during the cycling process. Therefore, the PNCs can be a suitable pseudocapacitive electrode material for LMA. The specific open surface area and porous structure of the PNCs were characterized by acquiring the nitrogen adsorption and desorption isotherms (Figure 1f). The isotherm was type‐II, corresponding to a macroporous structure based on the International Union of Pure and Applied Chemistry (IUPAC) classification. The pore size distribution plot in the inset of Figure 1f reveals more specific pore structures, where the pore volume gradually increases with the pore width on the nanoscale. The hierarchically nanoporous structure is consistent with the FE‐SEM images in Figure 1a. The specific surface area of PNC is ≈69.0 m^2^ g^−1^, in which most of the surface area arises from the outermost surfaces of the aggregated primary nanoparticles. X‐ray photoelectron spectroscopy (XPS) was used to probe the distinctive chemical structure of the PNCs (Figure 1g–i). The deconvoluted C 1s spectrum shows that sp^2^‐ and sp^3^‐hybridized carbon–carbon bonds form a major part of the structure, and numerous C—N and C—O bonds are present in the PNCs (Figure 1g). The oxygen groups can be divided into two main configurations, namely C═O and C—O bonds (Figure 1h). The atomic C/O ratio is ≈22.7, indicating that every 4–5 polyhexagonal carbon rings have one oxygen atom. Considering the well‐ordered graphitic structures, the oxygen functional groups were mainly located at the edge sites of the graphenic carbon planes. In the case of nitrogen dopants, pyridinic structures are a major configuration, and signals of pyridonic and quaternary nitrogen functional groups were also detected at 400 and 401.5 eV, respectively. Note that the atomic C/N ratio of the nitrogen dopants corresponds to ≈11.4, indicating that more than one nitrogen atom is doped on every two–three hexagonal carbon rings. It is well‐known that oxygen and nitrogen dopants can store lithium ions through a pseudocapacitive mechanism.^[^ ^33^, ^34^ ^]^ The surface‐induced reaction means that the top surfaces of the carbon materials are surrounded by chemisorbed lithium ions during the lithiation process. The effects of the pseudocapacitive heteroatom‐rich carbon materials on the LMA were investigated by comparing the electrochemical performance of the LMA with that of a reference anode comprising carbon black having few heteroatoms, but a similar specific surface area and morphological characteristics (see Figure S2, Supporting Information).

**Figure 2:**
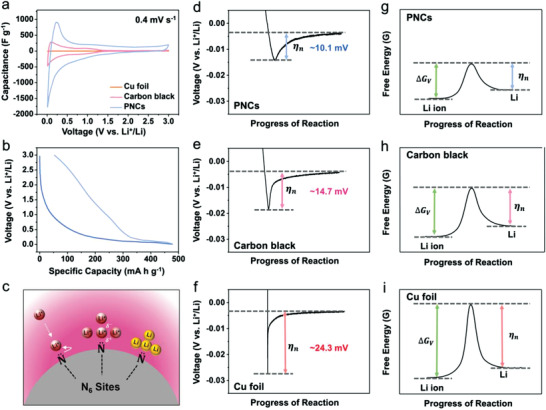
Electrochemical performances of Cu foil, carbon black, and PNCs as catalytic electrode materials for LMA. a) Cyclovoltammograms of Cu foil, carbon black, and PNCs at a scan rate of 0.4 mV s^−1^ and b) galvanostatic discharge/charge profiles of PNCs over voltage window of 0.01–3.0 V vs Li^+^/Li. c) Schematic image showing the catalytic effect of pyridinic nitrogen sites on PNC. Galvanostatic lithium metal deposition profiles of d) PNC, e) carbon black, and f) Cu foil at an areal current rate of 0.05 mA cm^−2^, and schematic images showing the relationship between the nucleation overpotential and free‐energy at g) PNC, h) carbon black, and i) Cu foil.

**Figure 3:**
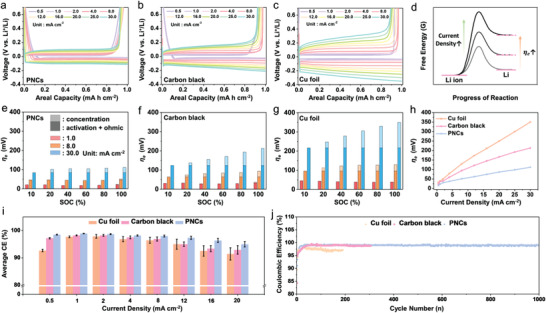
Kinetic performances and cycling stabilities of Cu foil‐, carbon black‐, and PNC‐based LMAs. Galvanostatic lithium metal deposition/dissolution profiles for a) PNCs, b) carbon black, c) Cu foil with a cut‐off capacity of 1.0 mA h cm^−2^ at different areal current rates. d) Schematic image showing the origin of *η*_e_ in the lithium metal deposition reaction process. Overpotential bar graphs for activation and ohmic overpotentials and concentration overpotential at e) PNC, f) carbon black, and g) Cu foil characterized at different areal current rates and different SoCs. h) *η*_e_ plots and i) CE bar graphs for Cu foil, carbon black, and PNCs according to current density. j) Cycling performance of Cu foil, carbon black, and PNCs with a cut‐off capacity of 1 mA h cm^−2^ at 1 mA cm^−2^ in half‐cell tests.

In the cyclic voltammograms acquired at a scan rate of 0.4 mV s^−1^ in the voltage window of 0.01‒3.0 V versus Li^+^/Li, the specific capacitance of the PNCs was much higher (≈3 and ≈100 times) than those of carbon black and the bare Cu foil, respectively, before lithium metal reduction (**Figure** **2**a). The similar capacitance gap between them was also confirmed in the dQ/dV curves (Figure S3, Supporting Information). During the discharge process (lithiation), the lithium cation concentration around the negative electrode increases due to the formation of electrochemical double layers, where a higher capacitance indicates that more cations accumulate on the electrode surfaces. Therefore, PNCs with high capacitance can facilitate faster and more stable lithium ion supply at high current rates. The galvanostatic discharge/charge profiles of the PNCs also reveal high specific lithium ion storage capacities, corresponding to ≈475 mA h g^−1^ (LiC_4.7_), where ≈300 mA h g^−1^ (LiC_7.5_) corresponds to lithium ions that are reversibly de‐lithiated with a linear voltage increase in the charge process (Figure 2b). The long‐range sloping voltage profile indicates that large numbers of lithium ions are chemisorbed on the surface of the PNCs, covering the entire surface of the PNCs in the lithiation process immediately before lithium metal nucleation. A high lithium ion concentration can facilitate lithium metal nucleation reactions when the voltage reaches a negative value. In particular, on the pyridinic nitrogen sites that are dominant on the surface of PNCs, strong chemical bonds can be formed between lithium ions and nitrogen owing to the high electronegativity of nitrogen. This can accelerate the formation of lithium metal nuclei via the catalytic effect, as shown in Figure 2c. Previously reported literatures highlighted the positive effects of the lithiophilic nitrogen functional groups, while the specific lithium nucleation mechanism has been poorly understood because of their non‐objective explanations.^[^ ^17^, ^40^, ^41^, ^42^ ^]^ In contrast, the schematic illustration suggested in Figure 2c clarifies the lithium metal nucleation behaviors on the pyridinic nitrogen sites of carbon‐based electrode materials. The catalytic effect of the PNCs was confirmed by determining the lithium metal nucleation overpotential (*η*_n_) in a galvanostatic discharge test at an areal current rate of 0.05 mA cm^−2^ (Figure 2d–f). The *η*_n_ value of the PNCs is ≈10.1 mV, which is much smaller than that (≈24.3 mV) of the Cu foil. This can be attributed to the large gap in the electrochemically active surface area (EASA). However, the higher *η*_n_ value (≈14.7 mV) of carbon black, which has a similar specific surface area, verifies the presence of a catalytic effect in addition to the EASA. The *η*_n_ value is affected by the free‐energy change per unit volume (*∆G*_V_) according to the following equation:

(1) $$\left|\eta_{n}\right|=\Delta G_{V}\;V_{m}/F$$

where *V*_m_ and *F* are the molar volume of lithium and the Faraday's constant, respectively.^[^ ^26^ ^]^ The relationship between the nucleation overpotential and free‐energy is schematically depicted in Figure 2g–i. On the pyridinic nitrogen sites of the PNCs, *∆G*_V_ can be significantly reduced by the catalytic effect, which means a large reduction in *η*_n_. More importantly, a much larger electrochemical overpotential (*η*_e_) was confirmed in the galvanostatic lithium metal deposition/dissolution profiles of carbon black and Cu foil acquired at different areal current rates (**Figure** **3**a–c). *η*_e_ affects the voltage gap between the Li metal deposition and dissolution profiles. Basically, *η*_e_ is composed of activation, ohmic, and concentration polarizations, where the ohmic and concentration polarizations have a critical effect on the rate capability at higher current rates. The schematic image in Figure 3d shows the specific origin of *η*_e_ in the reaction process. In the galvanostatic profiles of the Cu foil, a large voltage hysteresis between the discharge and charge profiles was observed (Figure 3c), where the hysteresis became significantly larger at higher areal current rates, indicating poor rate capabilities. In contrast, the voltage hysteresis was significantly reduced (by half) with carbon black (Figure 3b). Furthermore, the voltage hysteresis was much smaller for the PNCs than for the carbon black samples (Figure 3a). The change in the *η*_e_ values according to the areal current densities is shown in Figure 3h. The *η*_e_ values of the PNCs increased linearly from 15.4 to 112.0 mV at areal current densities between 0.5 and 30 mA cm^−2^, where these values were 1.9 and 3.1 times lower than those of the carbon black and Cu foil at 30 mA cm^−2^, respectively. One noteworthy result is the continuously increasing *η*_e_ values in the different states of charge (SoCs) for the Cu foil and carbon black. This is related to the successive increase in the overpotential in the galvanostatic profiles during lithiation (Figure 3b,c). The continuous voltage change (increasing *η*_e_) with the SoCs is mainly induced by concentration polarization, which originates from the deficiency of lithium ion charge carriers around the electrode surfaces. Comparison of the concentration polarization based on the respective *η*_e_ values for the Cu foil, carbon black, and PNCs clearly show that the PNCs afforded significantly lower concentration polarization, even at the higher current rate of 30 mA cm^−2^ (Figure 3e–g). Given that dendritic metal growth is favorable under diffusion‐controlled conditions, a lower concentration polarization is advantageous for dendrite‐mitigated lithium metal deposition/dissolution cycling. Hence, the PNCs afforded improved CE values and cycling performance.

The average CEs obtained with the Cu foil, carbon black, and PNCs were characterized at different areal current densities during the 20th to 100th cycles (Figure 3i). The average CEs were higher for the PNCs at all areal current densities, where the highest value of ≈99.0% was achieved at 1 mA cm^−2^, followed by a gradual decrease to ≈93.1% at areal current densities from 1 to 30 mA cm^−2^. These values are ≈0.8% and ≈1.3% higher than the maximum CEs of carbon black and Cu foil, respectively, and the gaps gradually increased with increasing areal current density. The 0.8% and 1.3% gaps of the CE values are very critical in the electrochemical performance of lithium metal batteries. During repetitive 100 cycles of the 99.0% PNCs, an excess lithium metal corresponding to 100% for the initial capacity is required to maintain a stable cycling process with full capacity retention, while the excess lithium metals of 180% and 230% for the initial capacities are needed for 98.2% carbon black and 97.7% Cu foil. The more excess lithium metal loading not only decreases the energy density of lithium metal batteries, but also increases the risk of safety hazard induced from the presence of excess lithium metals. Hence, the high average CE value of the PNCs can lead to higher energy performance in the relatively safe condition. In addition, the lithium metal deposition/dissolution cycles of the PNCs were well‐maintained, with higher CEs of ≈99.0% over 1000 cycles in the repetitive cycling test (Figure 3j). The cycling performance is better than that of Cu foil and carbon black in terms of both the average CE and long‐term stability. Ex situ FE‐SEM images of Cu foil and carbon black characterized after the cycling test in Figure 3j show that their active surfaces were clogged by large amount of side products (Figure S4a,b, Supporting Information). In contrast, the PNCs maintained the porous morphology even after the much longer cycles, supporting the superior cycling performance of PNCs (Figure S4c, Supporting Information). The results of the symmetric cell tests conducted with two identical PNC electrodes, including lithium metal at 2 mA h cm^−2^, further demonstrate the stable cycling performance of the PNCs over 1000 cycles (Figure S5, Supporting Information).

**Figure 4:**
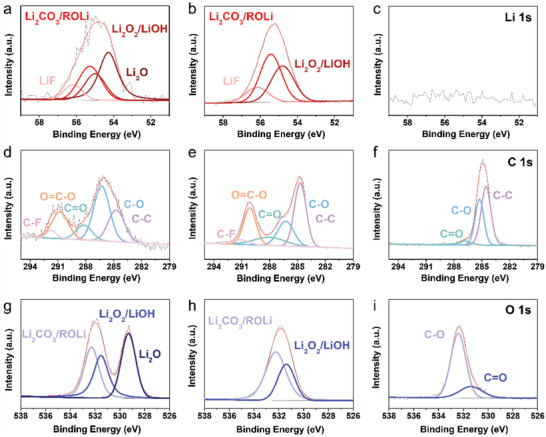
Ex situ XPS data for Cu foil, carbon black, and PNCs after 20th lithium metal deposition/dissolution cycle. Li 1s spectra of a) PNCs, b) carbon black, and c) Cu foil. C 1s spectra of d) PNCs, e) carbon black, and f) Cu foil. O 1s spectra of g) PNCs, h) carbon black, and i) Cu foil.

The electrochemical performance of LMA is closely related to the SEI layers formed on the surface of the electrode materials. Ex situ XPS analysis was conducted to define the chemical composition of the SEI layers on the different electrode materials after the 20th lithium metal deposition/dissolution cycle (**Figure** **4**). From the ex situ XPS Li 1s profile of the PNCs, several different types of L‐I‐SEI components such as Li_2_O, Li_2_O_2_, Li_2_CO_3_, and LiF were confirmed (Figure 4a), which is similar to the components observed with carbon black, except for Li_2_O (Figure 4b).^[^ ^43^, ^44^ ^]^ However, the lithium atomic ratio of the PNCs is ≈180% higher than that of carbon black, indicating the presence of more lithium‐rich components on the surface of the PNCs (Figure S6b,c, Supporting Information). The similar lithium atomic ratio between the PNCs and carbon black was maintained after the 100th lithium metal deposition/dissolution cycles (Figure S6e,f, Supporting Information). However, the L‐I‐SEI composition of the PNCs was changed with cycles, where the relative LiF to Li_2_O ratio was increased (Figure S7a, Supporting Information). In contrast, the carbon black showed the similar SEI composition after the 100th cycle (Figure S7b, Supporting Information). Interestingly, the SEI layers of the Cu foil had a low content of lithium components after the 20th cycle (Figure 4c). The surface of the Cu foil was fully covered with organic compounds composed mainly of C—C and C—O bonds and minor C═O bonds (Figure 4f). This is in contrast to the composition of the PNCs and carbon black, which consist of various chemical structures such as C—C, C—O, C═O, O—C═O, and C—F (Figure 4d,e).^[^ ^45^ ^]^ The atomic ratio of carbon was ≈72.5% for Cu foil, whereas the carbon content of the PNCs and carbon black was only ≈11.4% and ≈28.2%, respectively (Figure S6a–c, Supporting Information). This indicates that both the PNCs and carbon black have less organic SEI components on the surface of the electrode, and moreover, the PNCs have less than half of the carbon content of carbon black. To clarify the SEI layer of Cu foil, we further conducted the XPS characterization of the SEI layer‐formed Cu foil after Ar etching during 30 s (Figure S8, Supporting Information). In the ex situ XPS Li 1s depth profile, several lithium compounds such as Li_2_O_2_/LiOH, Li_2_CO_3_/ROLi, and LiF were detected (Figure S8a, Supporting Information), and the lithium atomic ratio was ≈18% (Figure S8b, Supporting Information). This result suggests that the surface organic SEI layers are considered quite thick because the XPS data without Ar etching shows only the outer organic layers. After the 100th lithium metal deposition/dissolution cycles, the outer SEI layer of Cu foil exhibited lithium‐including components such as Li_2_CO_3_/ROLi, Li_2_O_2_/LiOH, and LiF (Figure S7c, Supporting Information). However, the lithium contents were much lower than those of carbon black and PNCs (Figure S6d–f, Supporting Information). Additionally, the O 1s spectra confirm that the lithium‐rich inorganic component, Li_2_O, is a major constituent of the SEI layers on the PNCs, with other inorganic Li_2_O_2_/LiOH and Li_2_CO_3_/ROLi components after the 20th cycle (Figure 4g).^[^ ^43^, ^44^ ^]^ In contrast, no Li_2_O was observed on the SEI layers of carbon black, and the surface of the Cu foil was covered by organic components consisting of C—O and C═O bonds. The similar oxygen bonding configurations of the Cu foil, carbon black, and PNC were observed after the 100th cycle (Figure S7g–i, Supporting Information). Electrochemical impedance spectroscopy (EIS) tests were conducted to compare the surface film resistance (*R*_f_) of the different SEI layers after the 1st and 30th cycles (Figure S9, Supporting Information).^[^ ^32^ ^]^ The circuit simulation diagram obtained from the EIS profiles of three different electrodes exhibits that they are mainly composed of bulk solution resistance (*R*_e_) and *R*_f_ (Figure S9a, Supporting Information), where their *R*_e_ values were similar each other, while their *R*_f_ values were highly different (Figure S9b–d, Supporting Information). In the first cycle, the *R_f_* value for the PNCs was ∼30 Ω, which was reduced into ∼11 Ω after the cycling process by 30^th^ cycle. In contrast, the *R*_f_ values for the carbon black and Cu foil were increased during the ≈1st–30th cycles from ≈75 to ≈80 Ω and from ≈107 to ≈150 Ω, respectively. The enormous differences in the *R*_f_ values demonstrate that lithium ion transport occurs much faster in the L—I—SEI layers of the PNCs. Hence, the chemical structures and electrochemical performance of the SEI layers were highly affected by the electrode materials.

**Figure 5:**
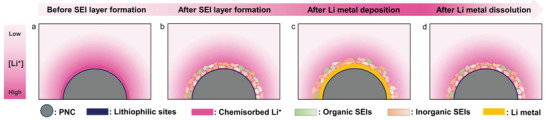
Schematic images showing effects of interfacial lithium ion concentration on PNC‐based LMA at different SoCs: a) before and b) after SEI layer formation, followed by c) lithium metal deposition and d) dissolution.

The schematic image shows the effects of the heteroatom‐rich pseudocapacitive electrode materials on formation of the SEI layer and on the lithium metal deposition/dissolution process (**Figure** **5**). In the lithiation process before SEI layer formation, EDL layers are formed around the electrode surfaces, where the nanostructured electrode materials can induce the formation of a thicker EDL layer with abundant lithium ion charge carriers (Figure 5a).^[^ ^46^ ^]^ In addition, numerous lithium ions are chemisorbed on the heteroatom active sites of the pseudocapacitive electrode materials, where the electrode surfaces can be completely covered by chemisorbed lithium ions.^[^ ^33^, ^34^ ^]^ As the electrode voltage decreased with lithiation, the electrolyte solution decomposed at the interface, and the resulting SEI materials were deposited on the electrode surface (Figure 5b).^[^ ^27^, ^28^, ^29^, ^30^ ^]^ Owing to the high lithium ion concentration at the interface, the irreversible reaction mainly produces lithium‐rich byproducts such as Li_2_O, Li_2_O_2_, and Li_2_CO_3_, which form highly ion‐conductive L–I–SEI layers. The thick EDLs and conductive L–I–SEI layers facilitated rapid and sustained lithium ion delivery over the entire electrode surface, leading to homogeneous lithium metal deposition (Figure 5c). In the reversible reaction process, the deposited lithium metal is dissolved in the EDLs of the electrolyte solution because the electric field is maintained after lithium metal stripping (Figure 5d).

To quantitatively analyze the preferential adsorption of Li^+^ on a pyridinic nitrogen doping site, density functional theory (DFT) calculations using Jaguar 8.4 were carried out.^[^ ^47^ ^]^ The non‐local van der Waals (vdW) density functional of the B3LYP‐D3 method along with the 6−31++G** basis set was used for electronic energy evaluation and geometry optimization to properly describe the vdW interactions.^[^ ^48^ ^]^ Two types of graphene quantum dots (GQDs) models having different edge structures (zigzag and armchair) were generated, where one carbon atom for each edge structure was replaced by nitrogen to introduce a pyridinic nitrogen doping site. Calculated binding energies of Li^+^ for pure GQDs models were similar values, −2.07 and −2.11 for zigzag and armchair, respectively (Figure S10, Supporting Information). For both cases, Li^+^ binding was preferred at the Pi‐electron‐rich C_6_ ring site rather than at the C—H edge site. However, binding energies were increased by −0.42 eV on both sites when the pyridinic nitrogen site was introduced, resulting from the direct interaction between Li^+^ and lone‐pair electrons at the pyridinic nitrogen. Stronger bindings of Li^+^ on pyridinic nitrogen at graphene edges could be the origin of lowered overpotential during the lithiation process. Furthermore, the presence of local Li^+^ binding sites could enhance the ionic interaction of the electrical double layer (EDL) due to the increased Li^+^ and counter‐anion concentrations in the EDL region. This is due to the additional chemisorbed Li^+^ in addition to the normal ionic distribution in the EDL region, which can be supported by the classical understandings of EDL behavior. When there is specific anion adsorption, the positive applied potential is countered by more than an equivalent charge of anions, thereby driving excess concentration of compensating cation in the EDL region.^[^ ^49^ ^]^

The PNC‐guided homogeneous lithium metal deposition/dissolution processes were confirmed by optical images and ex situ FE‐SEM analysis at different lithium metal deposition capacities (Figures S11–S14, Supporting Information). In the optical images of the reference Cu foil electrode before and after lithium metal deposition by 1 mA h cm^−2^ at an areal current rate of 4 mA cm^−2^, non‐uniform lithium metal growth was confirmed (Figure S11a,b, Supporting Information), and the ex situ FE‐SEM image characterized from the lithium metal deposited Cu foil showed formation of dendritic metals in the local area (Figure S11c, Supporting Information). When the carbon black electrode was used for the same ex situ tests, a metallic color sparsely covered in the entire area of the black color electrode after lithium metal deposition by 1 mA h cm^−2^, indicating that lithium metal deposition reactions happen relatively large surface area compared with the Cu foil electrode (Figure S11d,e, Supporting Information). The ex situ FE‐SEM image confirms that the lithium metal was filled in the whole area with no high aspect ratio metals (Figure S11f, Supporting Information). In contrast, the optical images of the PNCs showed no obvious color change after lithium metal deposition (Figure S11g,h, Supporting Information). In the ex situ FE‐SEM image, it was observed that the surface of the PNC was more densely covered by the deposited lithium metals (Figure S11i, Supporting Information). As the lithium metal deposition capacity increased, the macroporous structure was gradually filled with deposited lithium (Figure S12, Supporting Information). The cross‐sectional ex situ FE‐SEM images revealed that the internal pores were uniformly filled with lithium in a dense state after deposition at 1 mA h cm^−2^ (Figure S13, Supporting Information). In addition, the initial pore shape was recovered after the deposited lithium was fully dissolved, proving the uniform lithium metal deposition and the reversibility of this process (Figure S14, Supporting Information).

## 3 Conclusion

Effects of lithium ion concentration on the interface between electrode and electrolyte were studied by using different electrode materials such as Cu foil, carbon blacks, and PNCs. Because of an electric field in the lithiation process, the near surface of electrode materials was covered by EDL layers, wherein high surface area carbon materials were advantageous to form more lithium‐rich layers. In particular, the PNCs including multitudinous nitrogen and oxygen heteroatoms could store large numbers of lithium ions by chemisorption on their surfaces, which caused highly ionic‐conductive L–I–SEI layers. The PNC‐induced high lithium ion concentration and L–I–SEI layers guided homogeneous lithium metal deposition/dissolution cycles with high CEs of ≈99.0% over 1000 cycles. In addition, high rate capabilities with significantly low concentration polarization were achieved even at high areal current rates by 30 mA cm^−2^, which is in contrast to the Cu foil‐based LMA suffering from large concentration polarization. These results indicate that (1) lithium ion concentration on the interface is a key factor to determine electrochemical performances of LMA and (2) pseudocapacitive electrode nanomaterials are essential to develop high‐performance L–I–SEI layers and to concentrate large amount of lithium ion charge carriers on the electrode surface.

## 4 Experimental Section

### Preparation of PNCs

The synthesis of PNCs was performed using a direct current (DC) arc discharge process. The graphite powder (pure graphite > 99.9%, High Purity Chemicals) was densely packed in a graphite rod (7 mm in OD, 3 mm in ID, and 15 cm in length). Graphite powder‐filled rod and graphite plate were applied as anode and cathode for DC arc discharge synthesis, respectively. The arc chamber was evacuated and filled with nitrogen gas until a pressure of 300 torr. The applied current and voltage were set at 150 A and 30 V. The gap between the two electrodes was maintained at 2 mm during discharge. After arc synthesis finished, the temperature was cooled to room temperature. The PNCs were collected from the top of the arc chamber.

### Characterization

The material properties of the PNCs and carbon black were characterized using FE‐TEM (JEM2100F, JEOL, Japan), FE‐SEM (S‐4300SE, Hitachi, Japan), Raman spectroscopy (Renishaw InVia Raman spectrometer, Renishaw, U.K.), XRD (DMAX2500, Rigaku, Japan), XPS (PHI 5700 ESCA, Chanhassen, USA), and a porosimetry analyzer (ASAP2020, Micromeritics, USA). Electrochemical tests for the systems employing Cu foil, carbon black, and PNCs were conducted using an automatic battery cycler (Wonatech, South Korea) and CR2032‐type coin cells. Carbon black‐ and PNC‐based electrodes were prepared on Cu foil by coating with a slurry containing 90 wt% of the active materials and 10 wt% of polymer binder (polyvinylidene fluoride, Sigma–Aldrich, USA) in *N*‐methyl‐2‐pyrrolidone. Coin cells were assembled with an electrolyte of 1 m lithium bis(trifluoromethanesulfonyl)imide (LiTFSI, >99.95%, Sigma–Aldrich, USA) in a 1,3‐dioxolane/dimethyl ether mixture solution (1:1 v/v) containing 2 wt% LiNO_3_. Lithium foil (99.9%, Sigma–Aldrich, USA) was used as both the reference and counter electrodes, and a glass microfiber filter was used as a separator (GF/F; Whatman, UK). The working electrode was prepared by punching the bare Cu foil, carbon black‐coated Cu foil, or PNC‐coated Cu foil as 1/2 in. in diameter. The carbon black or PNC was loaded on the Cu foil with a loading density of ≈1 mg cm^−2^. EIS analysis was performed in the frequency range of 1 mHz to 0.1 MHz using an impedance analyzer (ZIVE SP2; WonATech). To prepare the symmetric cell composed of two same PNC electrodes including lithium metal of 2 mA h cm^−2^, the two PNC electrodes were prepared through a pre‐lithiation process in the respective half‐cells with a lithium metal foil. The symmetric cell composed of two same PNC electrodes including lithium metal of 2 mA h cm^−2^ was then assembled by using the Li/PNC electrodes extracted from the respective half‐cells.

## Conflict of Interest

The authors declare no conflict of interest.

## Supporting information

Supporting Information

## Data Availability

The data that support the findings of this study are available on request from the corresponding author.
