# Supplementary material for: Understanding the Effects of Interfacial Lithium Ion Concentration on Lithium Metal Anode
Source: Adv Sci (Weinh). 2021 Dec 22;9(6):2104145. doi: 10.1002/advs.202104145 (PMC8867159; doi:10.1002/advs.202104145)
Supplement: Supplementary file 1 — Supporting Information [file ADVS-9-2104145-s001.pdf]

WILEY-VCH

ADVANCED SCIENCE
Open Access

Supporting Information

for *Adv. Sci.*, DOI: 10.1002/advs.202104145

Understanding the effects of interfacial lithium ion concentration on lithium metal anode

*Jimin Park, Son Ha, Jae Young Jung, Jae-Hwan Hyun, Seung-Ho Yu, Hyung-Kyu Lim, Nam Dong Kim, and Young Soo Yun**

Supporting Information

Understanding for effects of interfacial lithium ion concentration on lithium metal anode

*Jimin Park, Son Ha, Jae Young Jung, Jae-Hwan Hyun, Seung-Ho Yu, Hyung-Kyu Lim, Nam Dong Kim, and Young Soo Yun**

Figure S1

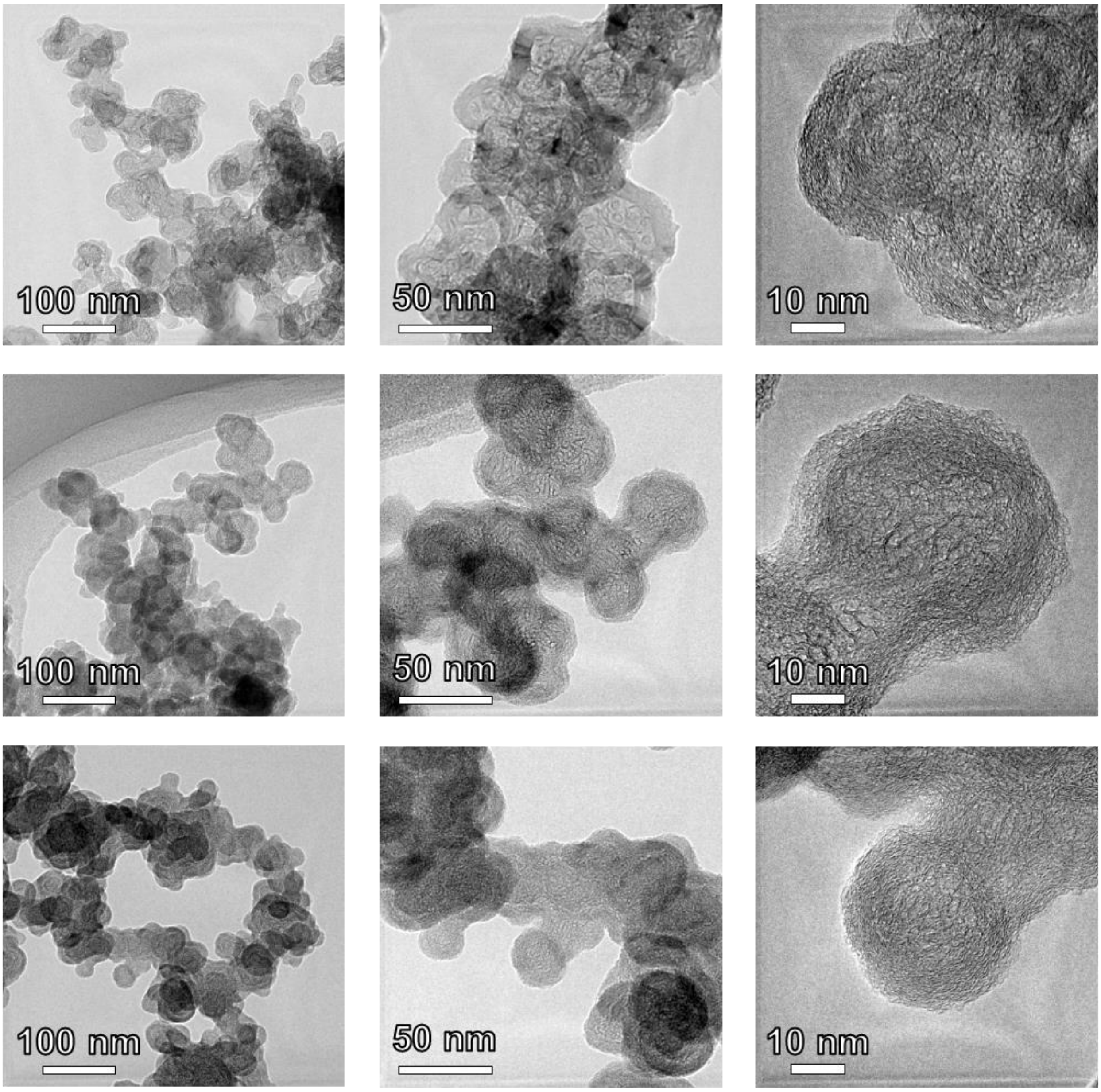

Figure S1. FE-TEM images of PNCs at different magnifications.

Figure S2

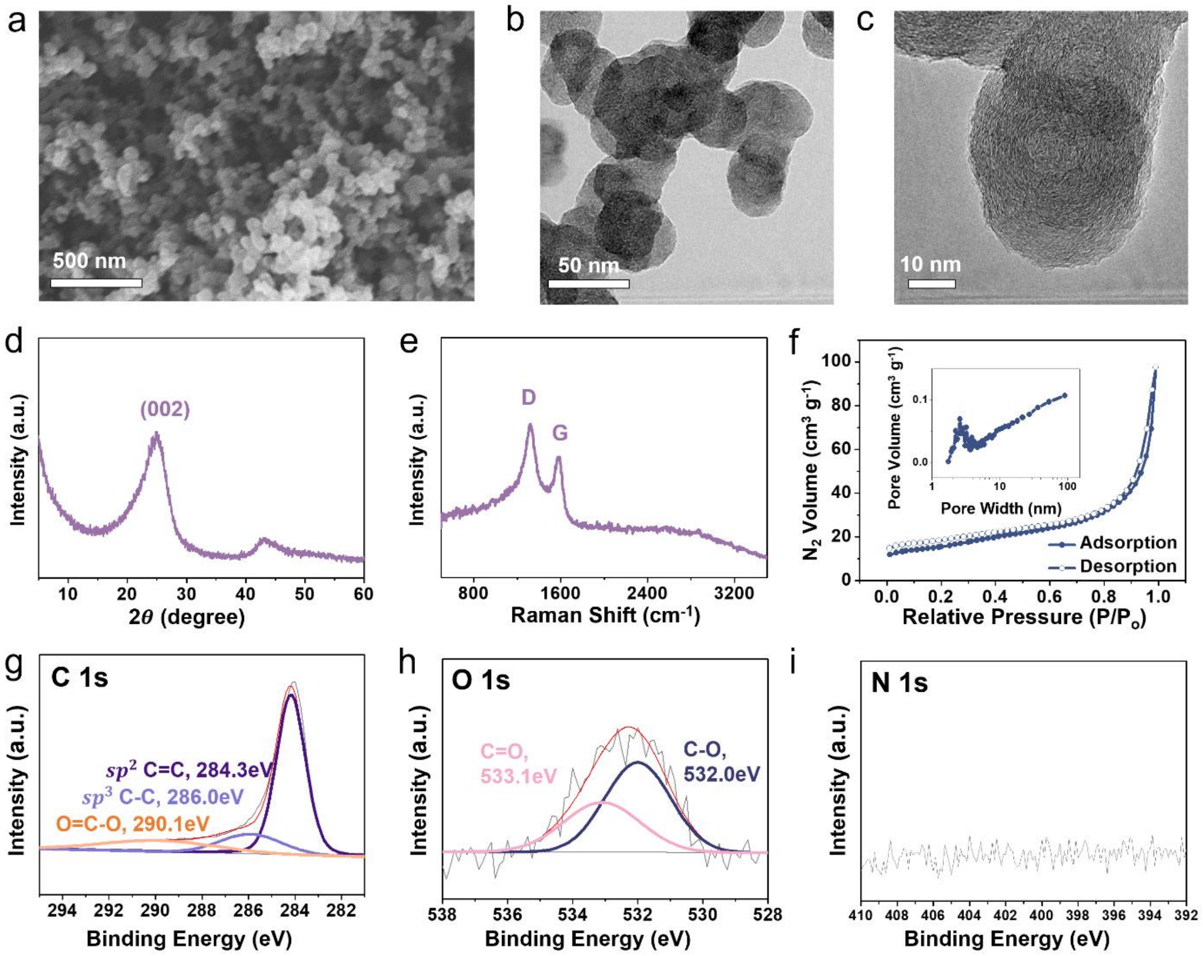

Figure S2. Materials properties of carbon blacks. (a) FE-SEM and (b, c) FE-TEM images at different magnifications. (d) XRD pattern, (e) Raman spectra, (f) nitrogen adsorption and desorption isotherms (inset shows pore size distribution), XPS (g) C 1s, (h) O 1s, and (i) N 1s profiles.

Figure S3

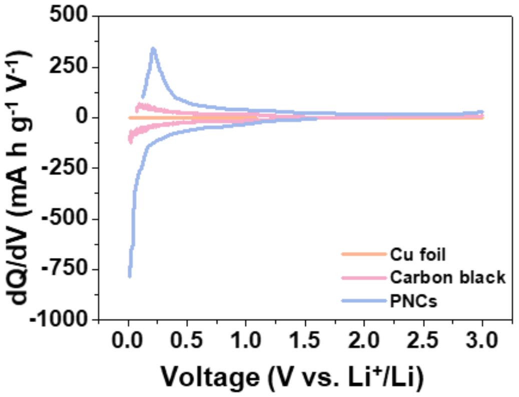

Figure S3. The dQ/dV curves of Cu foil, carbon black and PNCs obtained from their galvanostatic discharge/charge profiles at a current density of 1 A g^{-1} in the voltage window of 0.01–3.0 V vs. Li^{+}/Li.

Figure S4

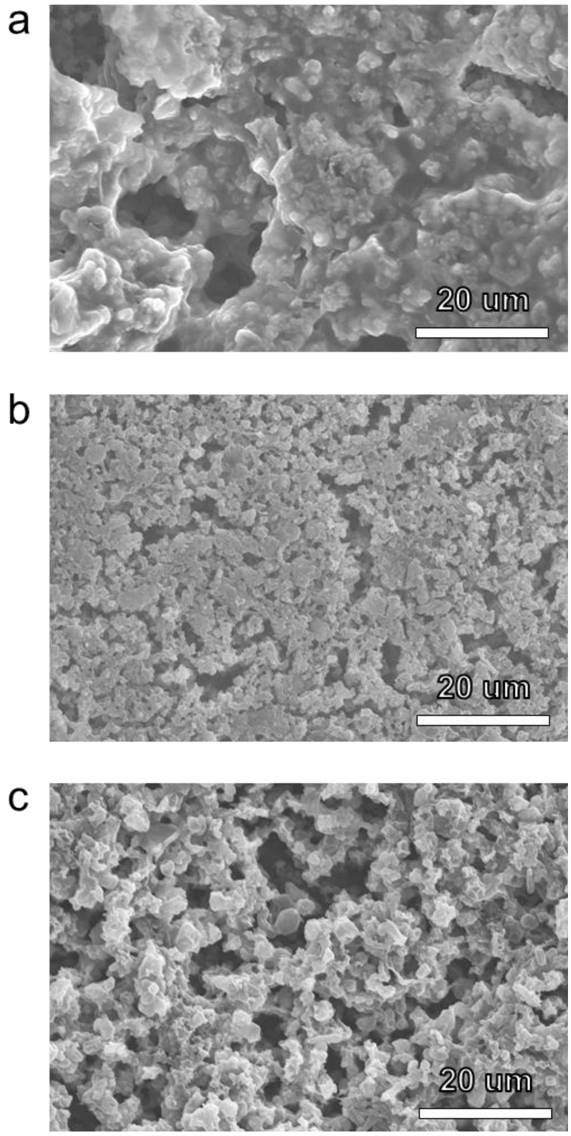

Figure S4. Morphologies of (a) Cu foil, (b) carbon black, and (c) PNCs characterized by ex situ FE-SEM observations after the cycling process of Fig. 3j.

Figure S5

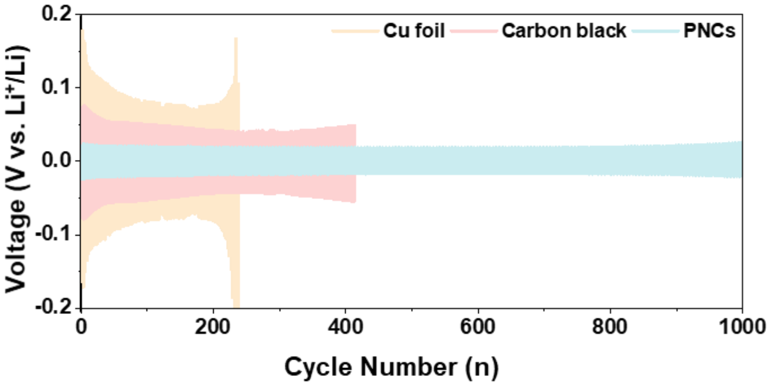

Figure S5. Galvanostatic charge/discharge profiles obtained from symmetric cells composed of two same PNC, Carbon black or Cu foil electrodes including lithium metal at 2 mA h cm^{-2}.

Figure S6

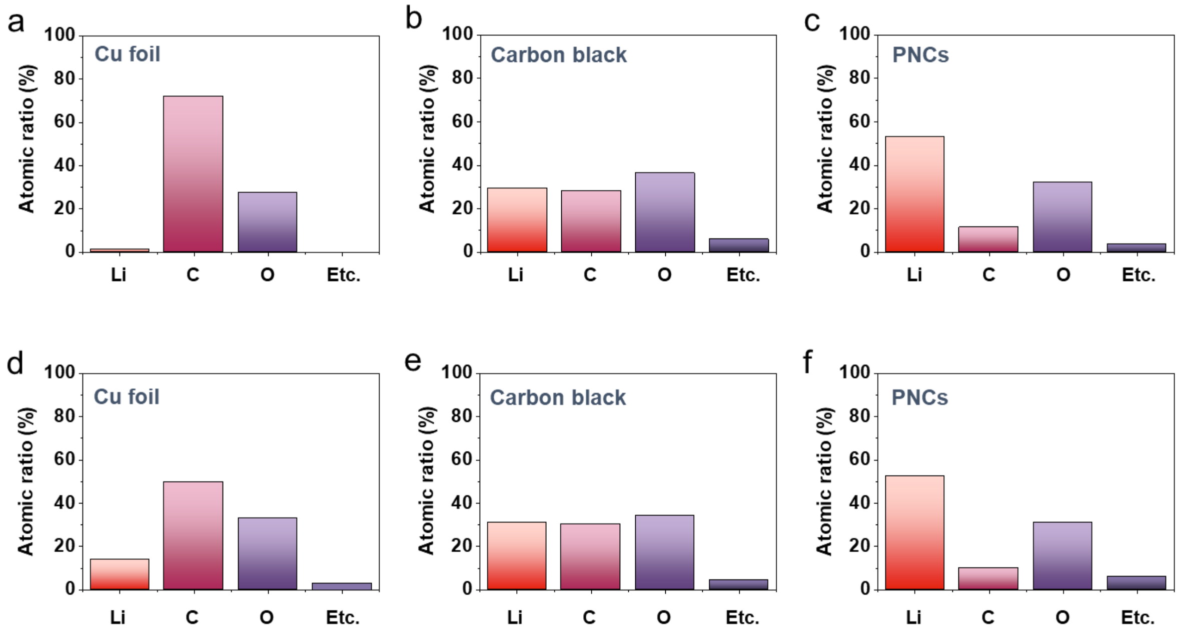

Figure S6. Atomic ratio bar graphs of (a) Cu foil, (b) carbon black, and (c) PNC for Li, C, O and Etc (N, S, F) characterized from ex situ XPS analysis after 20th cycle, and those of (d) Cu foil, (e) carbon black, and (f) PNC after 100th cycle.

Figure S7

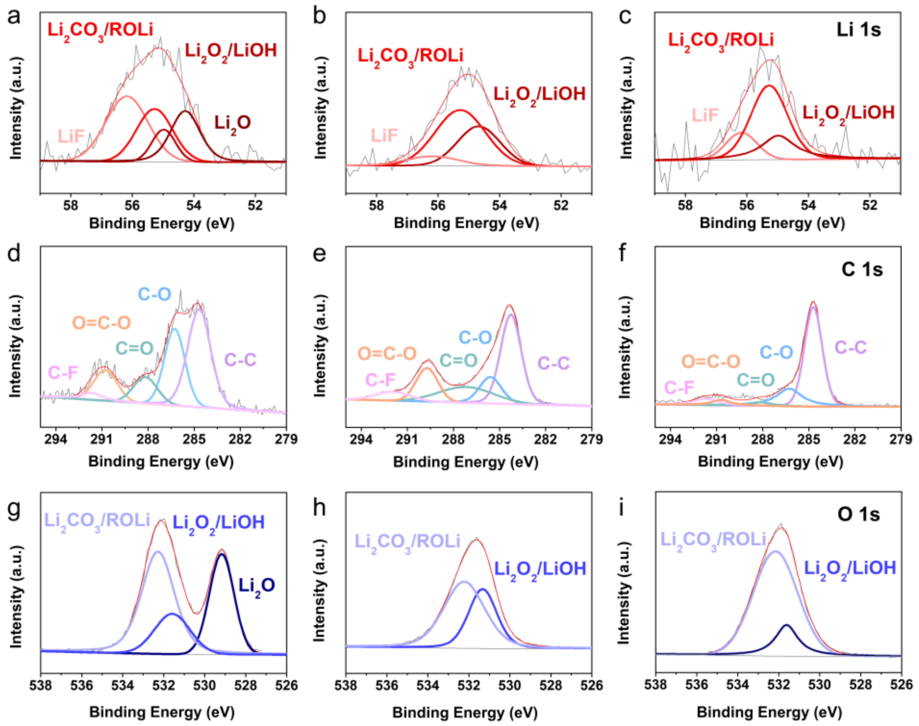

Figure S7. Ex situ XPS data for Cu foil, carbon black, and PNCs after 100th lithium metal deposition/dissolution cycle. Li 1s spectra of (a) PNCs, (b) carbon black, and (c) Cu foil. C 1s spectra of (d) PNCs, (e) carbon black, and (f) Cu foil. O 1s spectra of (g) PNCs, (h) carbon black, and (i) Cu foil.

Figure S8

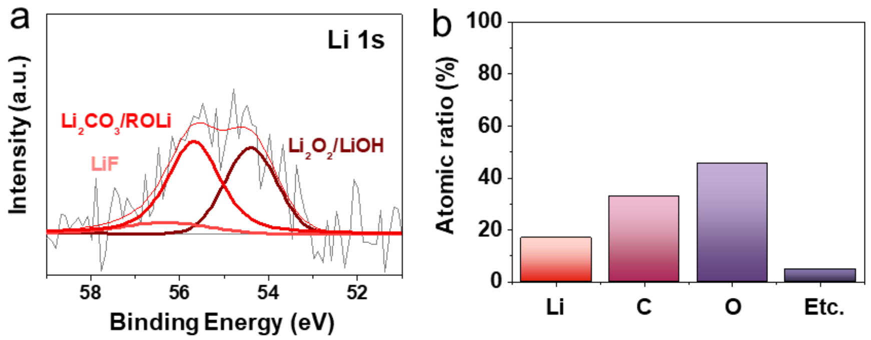

Figure S8. (a) Ex situ XPS Li 1s depth profile of the SEI-layer-formed Cu foil characterized after Ar etching during 30 s, and (b) bar graphs for relative atomic ratio of Li, C, O and etc.

Figure S9

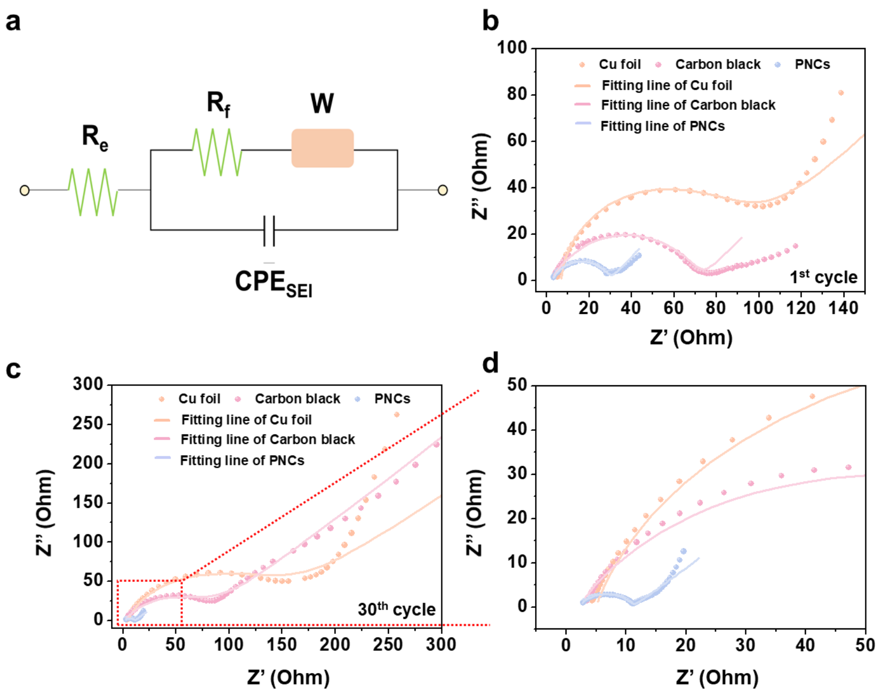

Figure S9. (a) The circuit simulation diagram obtained from the EIS profiles of Cu foil, carbon black, and PNC, and EIS data including the output impedance spectra of the fitted circuit of Cu foil, carbon black, and PNC characterized after (b) 1st and (c, d) 30th cycles.

Figure S10

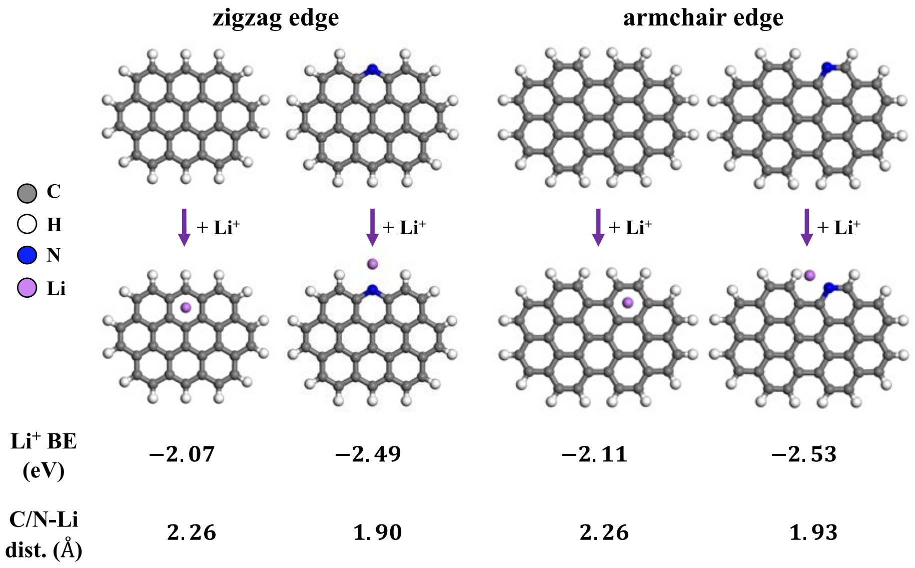

Figure S10. Calculated Li^+ binding structures and energies for pure graphene edge and pyridinic nitrogen-doped graphene edge sites.

Figure S11

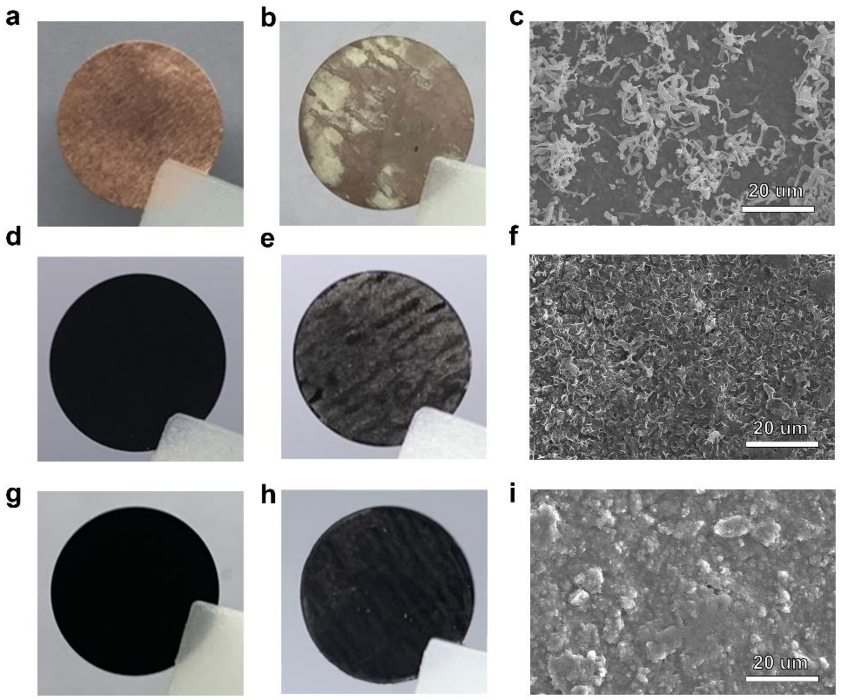

Figure S11. Optical images of bare (a) Cu foil, (d) carbon black and (g) PNC, and ex situ (b) Cu foil, (e) carbon black and (h) PNC after lithium metal deposition by 1 mA h cm^{-2} at an areal current density of 4 mA cm^{-2}. Ex situ FE-SEM images of (c) Cu foil, (f) carbon black and (i) PNC characterized from (b), (e) and (h), respectively.

Figure S12

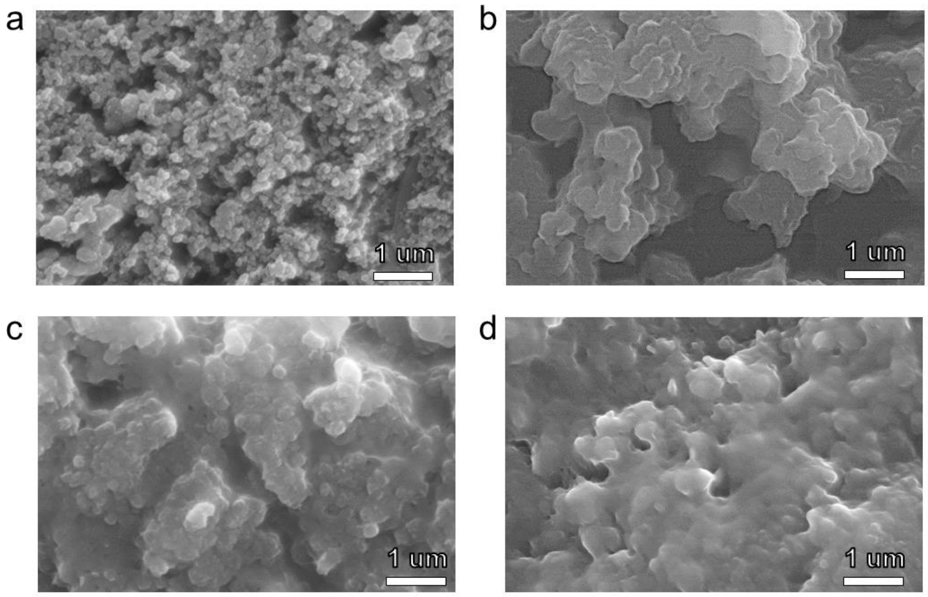

Figure S12. Ex situ FE-SEM images of PNCs characterized after lithium metal deposition of (a) 0, (b) 0.2, (c) 0.5, and (d) 1.0 mA h cm^{-2}.

Figure S13

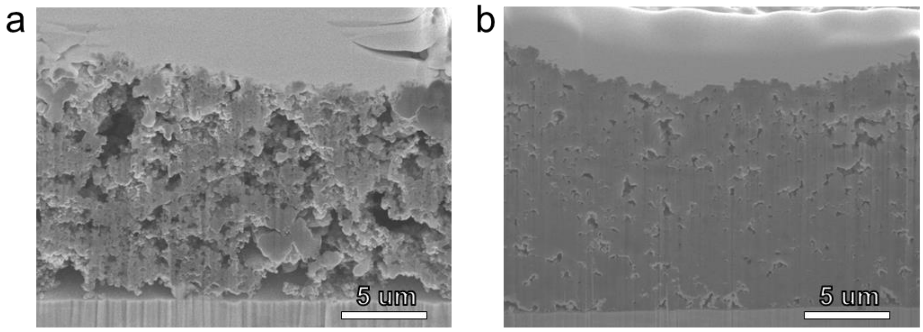

Figure S13. Cross-sectional ex situ FE-SEM images of (a) pristine PNCs and (b) PNCs including lithium metal of 1.0 mA h cm^{-2}.

Figure S14

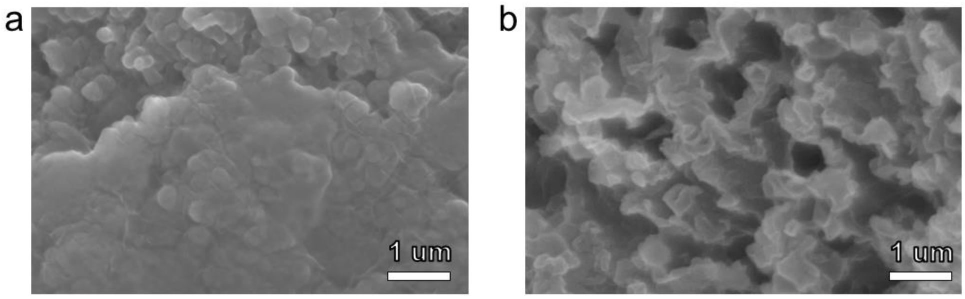

Figure S14. Ex situ FE-SEM images of (a) PNCs after lithium metal deposition of 1.0 mA h cm^{-2} and (b) followed by lithium metal stripping.
